# Supplementary material for: Photooxidation driven formation of Fe-Au linked ferrocene-based single-molecule junctions
Source: Nat Commun. 2024 Feb 16;15:1439. doi: 10.1038/s41467-024-45707-z (PMC10873316; doi:10.1038/s41467-024-45707-z)
Supplement: Supplementary file 1 — Supplementary Information [file 41467_2024_45707_MOESM1_ESM.pdf]

SUPPLEMENTARY INFORMATION

Photooxidation Driven Formation of Fe-Au Linked Ferrocene-Based Single-Molecule Junctions

Woojung Lee[1], Liang Li[1], María Camarasa-Gómez,[2] Daniel Hernangómez-Pérez,[2] Xavier Roy[1], Ferdinand Evers,[2] Michael S. Inkpen,[3]* Latha Venkataraman[1,4]*

[1]*Department of Chemistry, Columbia University, New York, New York 10027, United States*
[2]*Institute of Theoretical Physics, University of Regensburg, 93040, Regensburg, Germany*
[3]*Department of Chemistry, University of Southern California, Los Angeles, California, 90089, United States*
[4]*Department of Applied Physics and Applied Mathematics, Columbia University, New York, New York 10027, United States*

Email: lv2117@columbia.edu; inkpen@usc.edu, ferdinand.evers@physik.uni-regensburg.de

Contents:

Supplementary Note 1 – Cyclic Voltammetry (CV)

Supplementary Note 2 – Additional Conductance Data

Supplementary Note 3 – DFT Calculations

Supplementary Method – Synthetic Details and NMR spectra

Supplementary References

Supplementary Note 1 - Cyclic voltammetry (CV)

We characterize the electrochemical properties of ferrocene derivatives, **1-3**, with in situ two-electrode cyclic voltammetry (CV) measurements. These measurements were performed in a two-terminal electrochemical cell using the STM-BJ wax coated gold tip and substrate.[9] 50 μM solutions of each derivative in propylene carbonate (PC) were used. The distance between two gold terminals was maintained at around 100 nm during the measurement. The measured data was obtained by recording the current flowing into the tip as the voltage was swept linearly the substrate. The redox reaction of each derivative was reversible over at least 5 cycles.

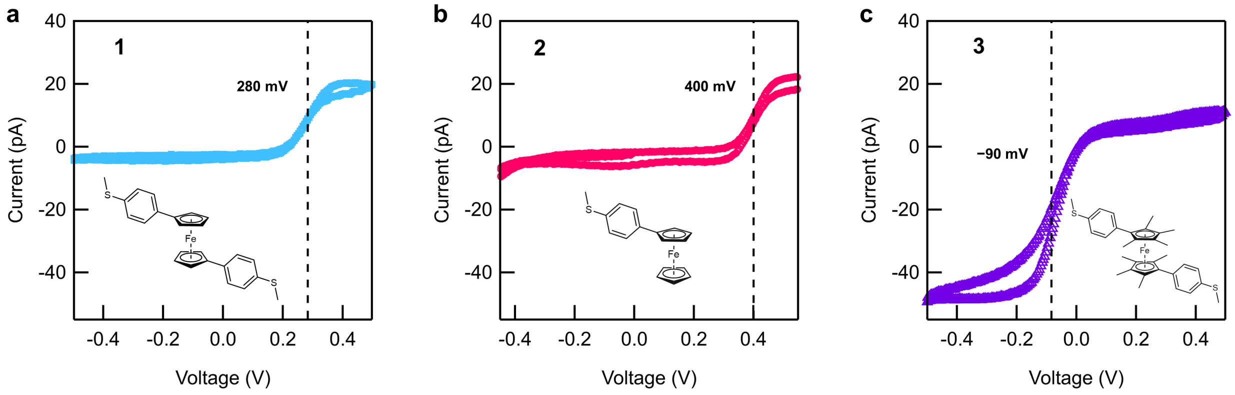

Supplementary Fig. 1. *In situ* cyclic voltammetry (CV) measurements of ferrocene derivatives, **1-3**. The determined redox potentials of **1-3** are 280 mV, 400 mV, and − 90 mV, respectively.

Supplementary Note 2 - Additional Conductance Data

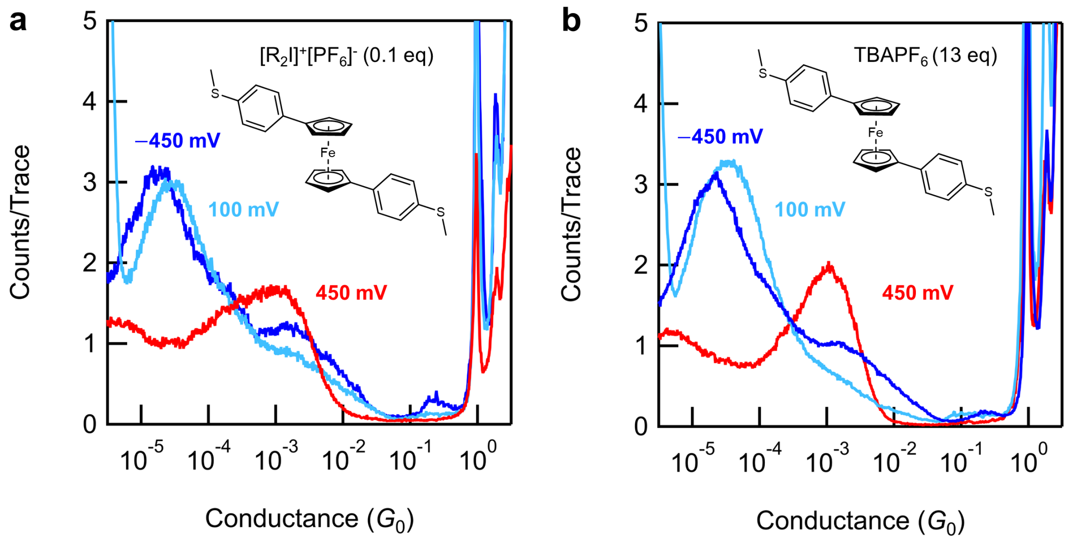

Supplementary Fig. 2. (a) One-dimensional (1D) conductance histograms of **1** with ~ 0.1 equiv. of $[R_2I]^+[PF_6]^-$ in propylene carbonate (PC), keeping the same condition as the photoredox measurements. The results are the same as the measurement without the anion in Fig. 2a, implying that the $[PF_6]^-$ does not affect the junction formations proposed here. (b) 1D conductance histograms of **1** with an excess of $[PF_6]^-$ (13 equiv.). Given that a high concentration of iodine hinders the formation of molecular junctions, we utilized $TBAPF_6$ to examine the effect of $[PF_6]^-$. Again, the results are the same as those made without the counterion. Therefore, we can exclude the possibility that we cannot form a junction in the presence of $[PF_6]^-$.

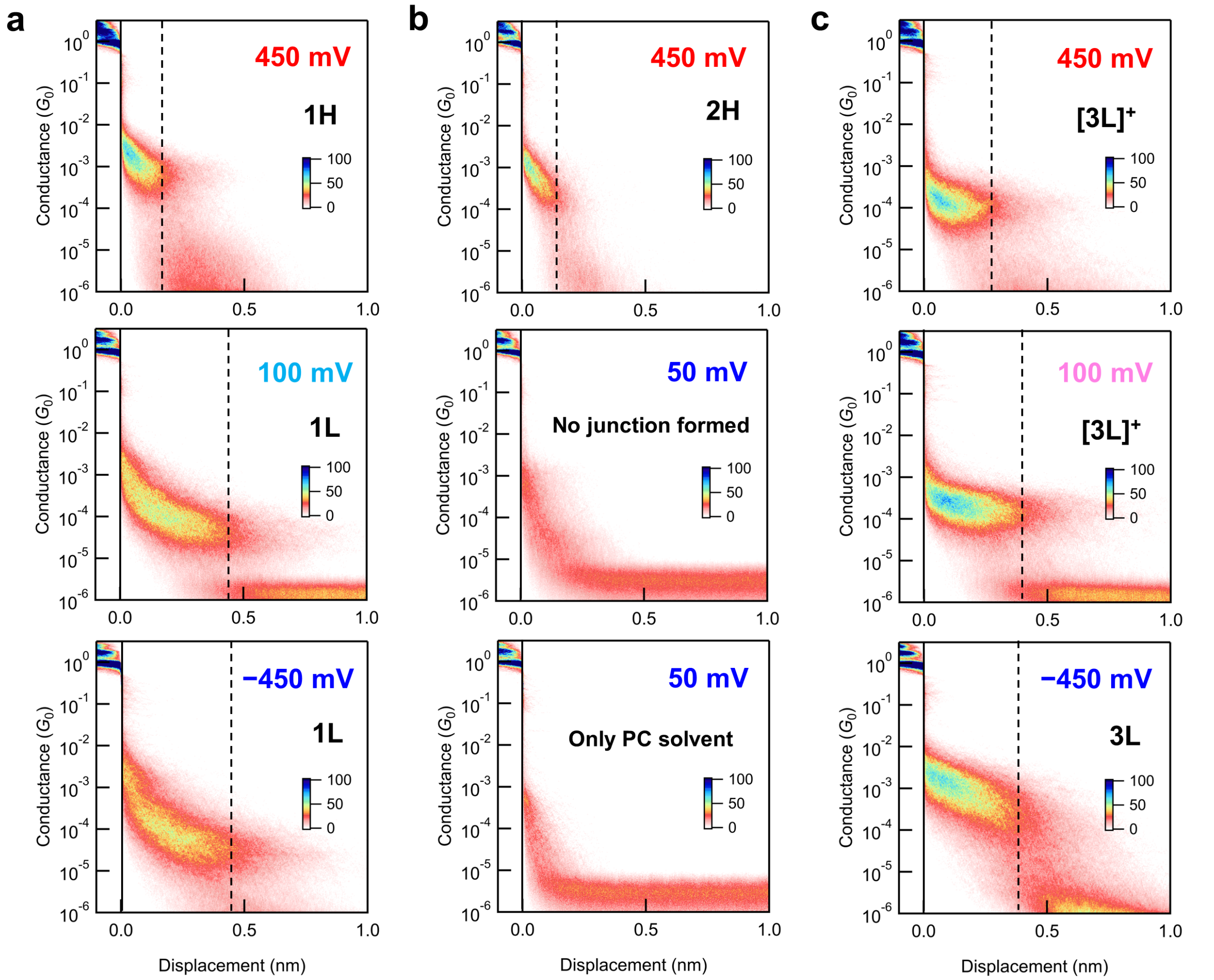

Supplementary Fig. 3. Two-dimensional (2D) conductance-displacement histograms of **1** (a), **2** (b), and **3** (c) in PC at different bias voltages. Each molecular junction geometry is indicated as **1H**, **1L, 2H** and **3L**. (a) The oxidized **1** at 450 mV forms a short molecular junction of **1H**, while neutral states at 100 and −450 mV form a longer junction, **1L**. (b) 2D histogram of **2** in its neutral state at 50 mV is almost identical with that of PC solvent measurement without molecule **2**, indicating there is no molecular junction formed. (c) Both oxidized and neutral states of **3** form the same junction geometry of **3L**. Given that **1** and **2** exhibit elevated conductance at more positive bias voltages, the electron transport through the molecular junctions is dominated by HOMO resonance. Conversely, **3** shows higher conductance at more negative voltages, suggesting the LUMO-dominant electron transport. During the STM-BJ measurements, the junction length of **3L** shortens as the applied bias increases; this is consistent with our previous report about LUMO-conducting molecules in polar solvents.[10]

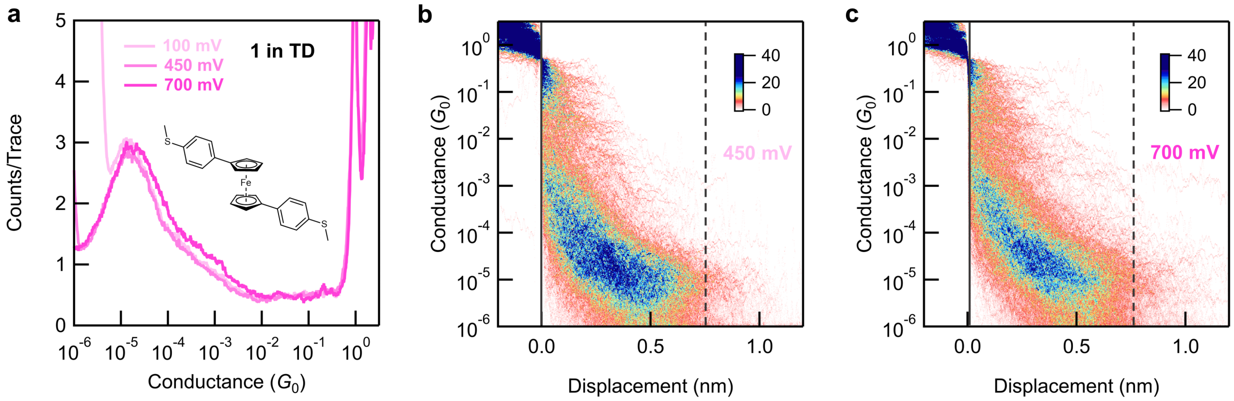

Supplementary Fig. 4. (a) 1D conductance histogram of **1** in tetradecane (TD) at 450 mV and 700 mV. (b-c) 2D conductance-displacement histograms at 450 mV and 700 mV, respectively. The solution was prepared on the same day.

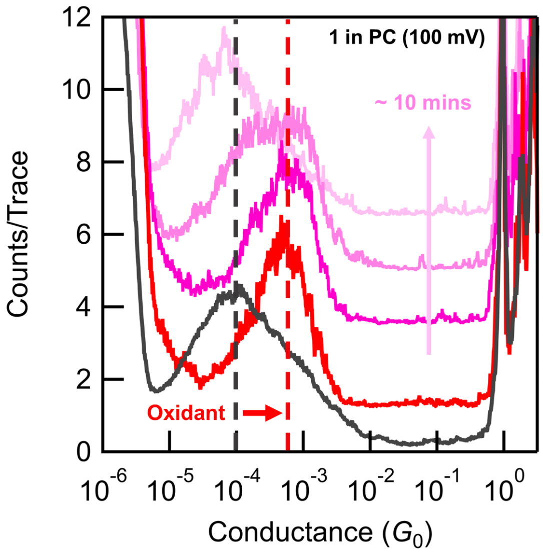

Supplementary Fig. 5. 1D conductance histogram of **1** at 100 mV. After adding oxidant, a 50 μM solution of tris(4-bromophenyl)ammoniumyl hexachloroantimonate (BAHA) in PC, the conductance peak shifts up (dark gray to red). As a low bias (100 mV) is applied continuously during the measurement, the conductance returns to the lower regime after minutes.

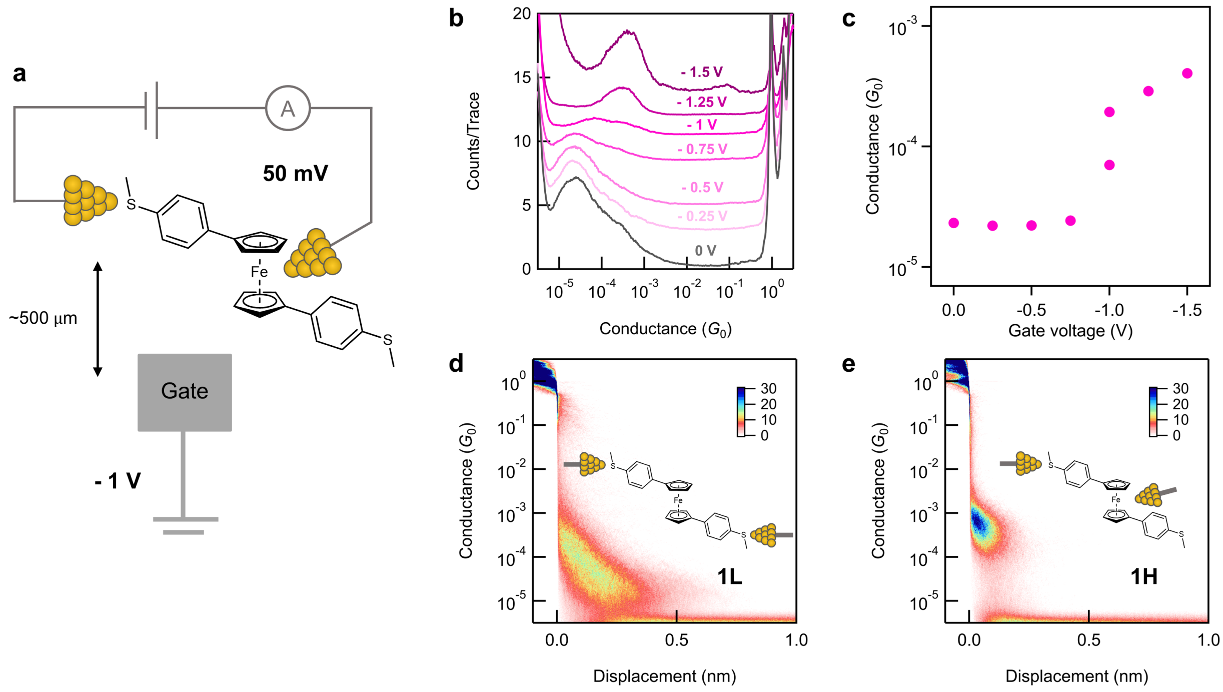

Supplementary Fig. 6. (a) Schematic of three-electrode STM-BJ measurements, employing a Pt wire gate electrode that is in the solution of **1** with $TBAPF_6$ as electrolyte. The gate electrode functions as both the counter and reference electrodes, while the gold tip and substrate serve as the working electrodes. To ensure that the applied gate potential drops across the working electrodes, a small tip/substrate bias of 50 mV is applied, which is significantly lower than the redox potential of **1**. As the gating voltage becomes more negative, more molecules in the solution are oxidized at the tip and substrate electrodes, leading the formation of **1H** geometry. (b) Overlaid 1D conductance histograms of **1** at different gate potential voltages. (c) Gaussian-fitted conductance values at each gate voltage. (d-e) 2D conductance-displacement histograms at gate voltage of 0 V and -1.5 V, respectively.

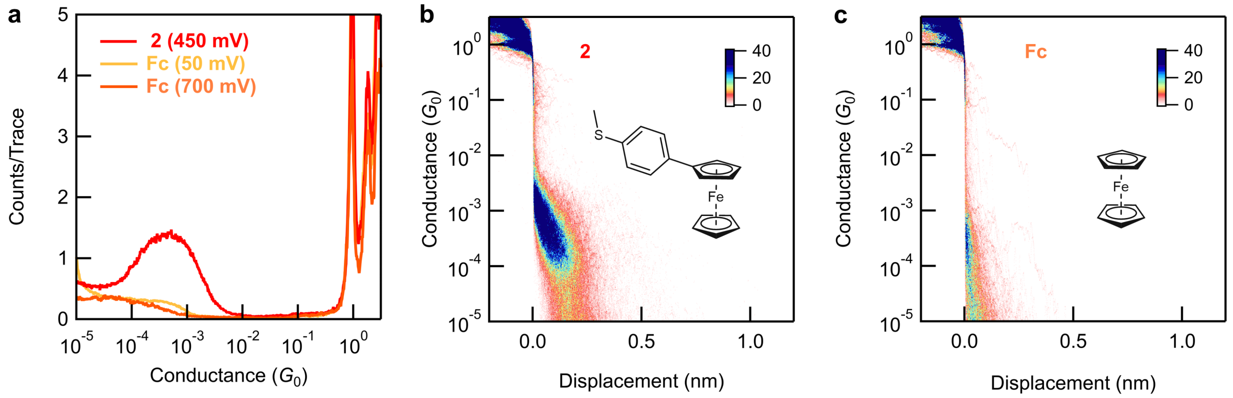

Supplementary Fig. 7. (a) 1D conductance histogram of **2** and ferrocene (Fc) solutions in PC at 450 mV and 50 mV applied bias. (b and c) The resulting 2D conductance-displacement histograms at 450 mV. Any molecular junction is not formed in the ferrocene solution in the experimental conditions used here. The determined redox potential of ferrocene is 420 mV in this setup.

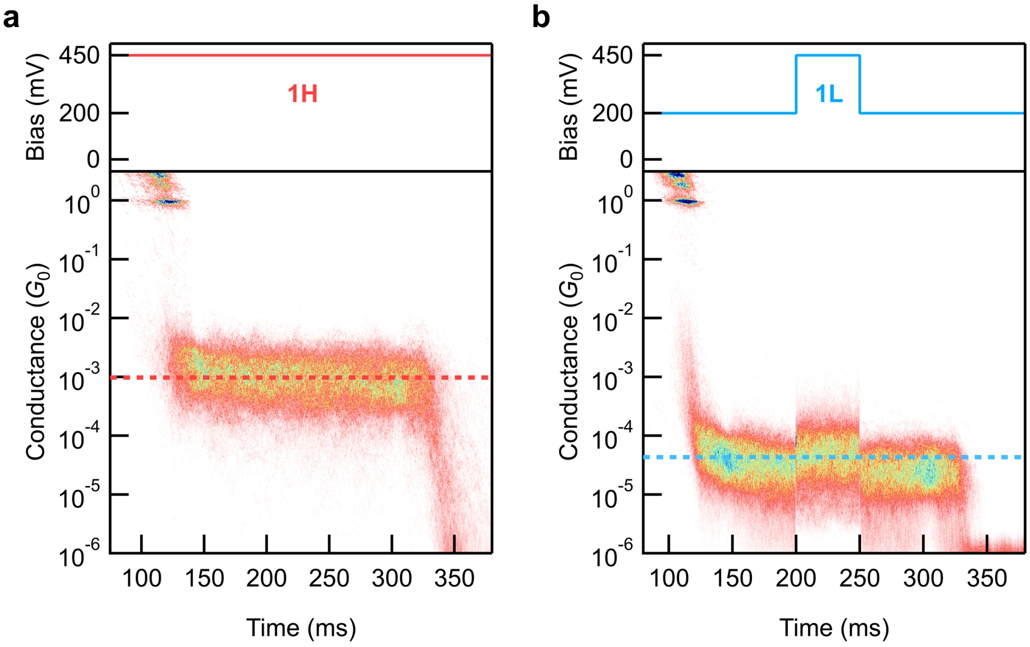

Supplementary Fig. 8. STM-BJ holding measurements. Top: The switching bias ramp applied across molecular junctions as a function of time. Bottom: 2D conductance-time histograms obtained for **1** using different bias ramps. Two different junction geometries are formed by controlling the oxidation state of **1** with the applied voltage: (a) **1H** at 450 mV and (b) **1L** at 200 mV. The most probable measured conductance of both geometries at 450 mV is indicated with the dashed line.

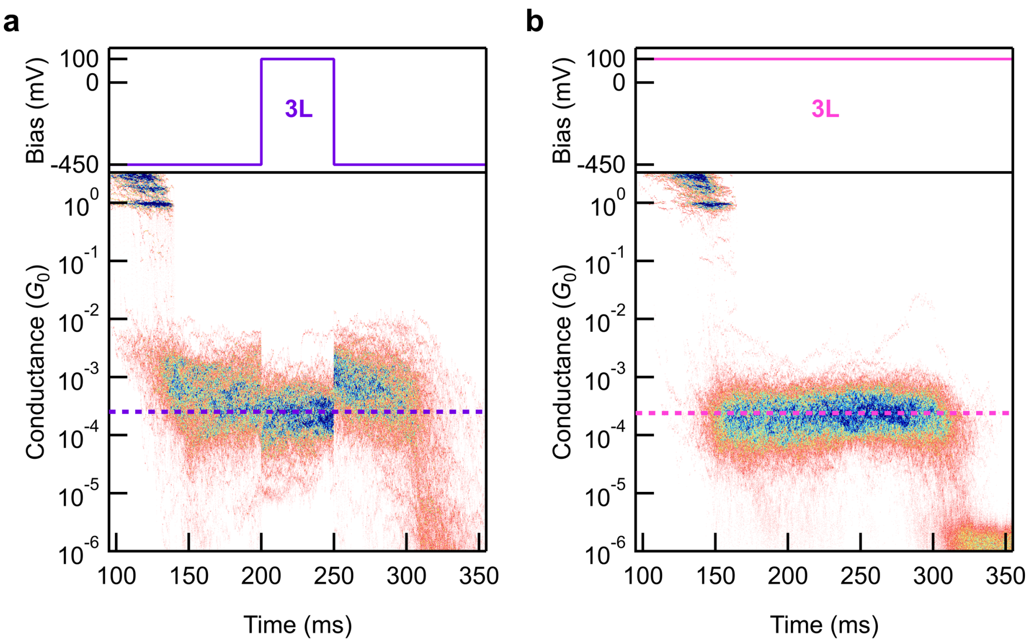

Supplementary Fig. 9. STM-BJ hold measurements with **3**. *Top*: Switching bias applied across molecular junctions. *Bottom*: 2D conductance-time histogram. The same **3L** geometry is formed with two different applied bias voltages of –450 mV (a) and 100 mV (b). The measured conductance of **3L** junction at 100 mV is highlighted with the dashed line.

Supplementary Note 3 - DFT calculations

Simulated space-filling (CPK) models

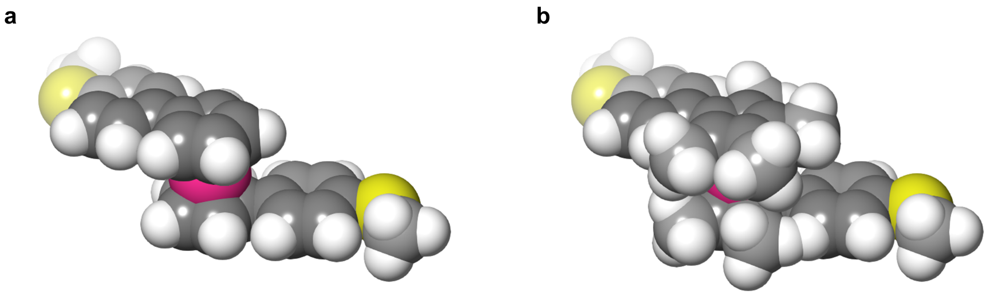

Supplementary Fig. 10. Space-filling (CPK) models of (a) **1** and (b) **3**. Grey, white, yellow, and magenta spheres represent C, H, S, Fe atoms, respectively. The methyl groups sterically block the ferrocene iron center from the approaching tip gold atom, prohibiting bond formation between Fe and Au.

Electronic properties of Fe-Au bond

As discussed in the main paper, we determine the binding energy between Fc and the Au contact using the DFT methods detailed below. The binding energy is determined with the van der Waals (vdW) correction.[11]

$$E_{bind} = E_{DFT} + E_{vdW} \quad (1)$$

where E_{DFT} is the self-consistent Kohn-Sham energy and E_{vdW} is an empirical vdW energy given by

$$E_{vdW} = -s_6 \sum_{i=1}^{N-1} \sum_{j=i+1}^{N} \frac{C_6^{ij}}{R_{ij}^6} f_{dmp}(R_{ij}). \quad (2)$$

s_6 is a global scaling factor, N is the number of atoms in the system, C_6^{ij} ($= \sqrt{C_6^i \times C_6^j}$) indicates the dispersion coefficient for atom pair (ij), R_{ij} is the distance between the atom pair, and f_{dmp} is a damping function for avoiding near-singularities for small R.

$$f_{dmp}(R_{ij}) = \frac{1}{1+e^{-d(\frac{R_{ij}}{R_r}-1)}} \quad (3)$$

where R_r is the sum of atomic vdW radii ($r_i + r_j$) and d is a damping factor. During the computation of the vdW energy, we exclude the intramolecular vdW interactions within Fc and Au cluster. All parameters used here are given in Supplementary Table 1.

	C_6	r
**C**	1.750	1.452
**H**	0.140	1.001
**Fe**	10.80	1.562
**Au**	40.620	1.772

Supplementary Table 1. C_6 parameters ($J \cdot nm^6 mol^{-1}$) and van der Waals Radii, r (Å). The values of s_6 and d are 0.75 and 20.0 Å, respectively.[11-13]

Here, two distinct geometries are considered for comparative analysis: 1) bonding between ferrocene Fe center and Au cluster, and 2) bonding between π-orbital of Cp ring and Au cluster, shown in Supplementary Fig. 11. The binding energies (E_{bind}) are determined as 0.80 eV and 0.29 eV, respectively, with the vdW correction. We also calculate the frontier molecular orbitals (MOs) and molecular conductance orbitals (MCOs) of **1-3** in the molecular junctions (Supplementary Fig. 12-14). We note that the angle between two Cp ring planes of ferrocene in the calculation is 22.6º in agreement with previously reported results.[14] This tilt angle inhibits ferrocene iron center from binding with two Au electrodes on diametrically opposite sides. These calculations are consistent with the experimental results that a ferrocene by itself does not form a molecular junction between two Au electrodes (Supplementary Fig. 7).

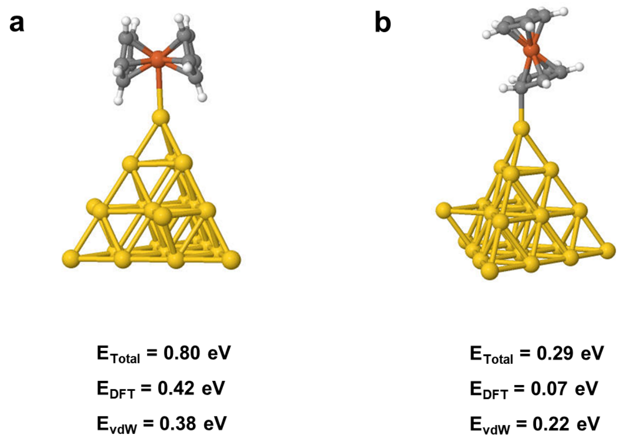

Supplementary Fig. 11. Calculated binding energies of (a) Fe-Au bonding between ferrocene and Au cluster, and (b) Cp-Au bonding via the vdW interaction between the Cp ring and Au cluster. The self-consistent Kohn-Sham energies (E_{DFT}) were obtained from the energy difference between the total energy of the bound geometry and the sum of the energies of the isolated molecules and the gold cluster. The bond lengths in each geometry are (a) 3.01 Å (Fe-Au), and (b) 2.26 Å (C-Au).

Calculated molecular orbitals of 1, 2 and 3

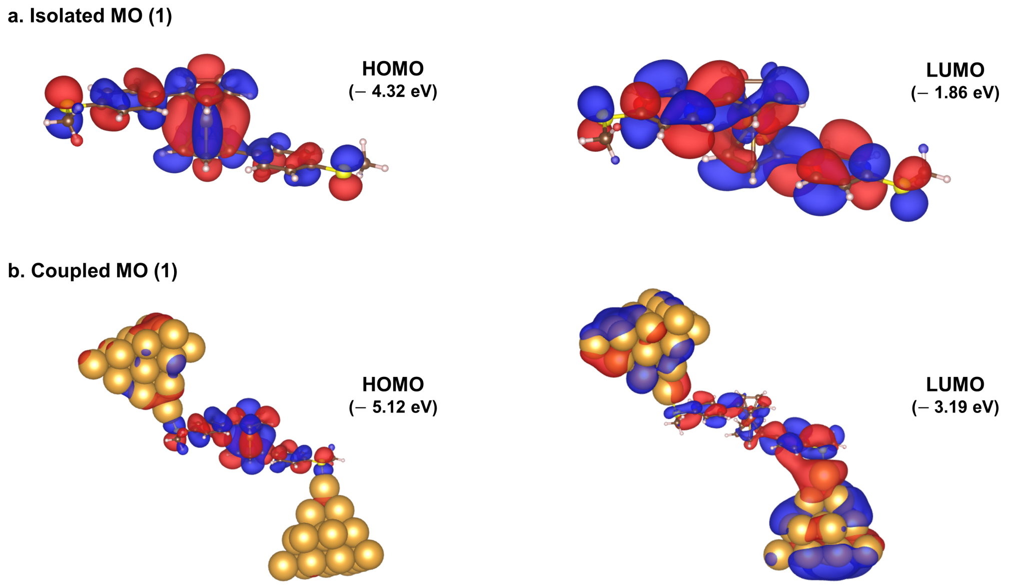

Supplementary Fig. 12. (a) Molecular orbitals of isolated molecule **1L**. (b) Scattering states of the **1L** junction, that contribute to transmission at E_F. Each frontier orbital energy is calculated relative to the vacuum level. E_F is – 4.49 eV relative to vacuum.

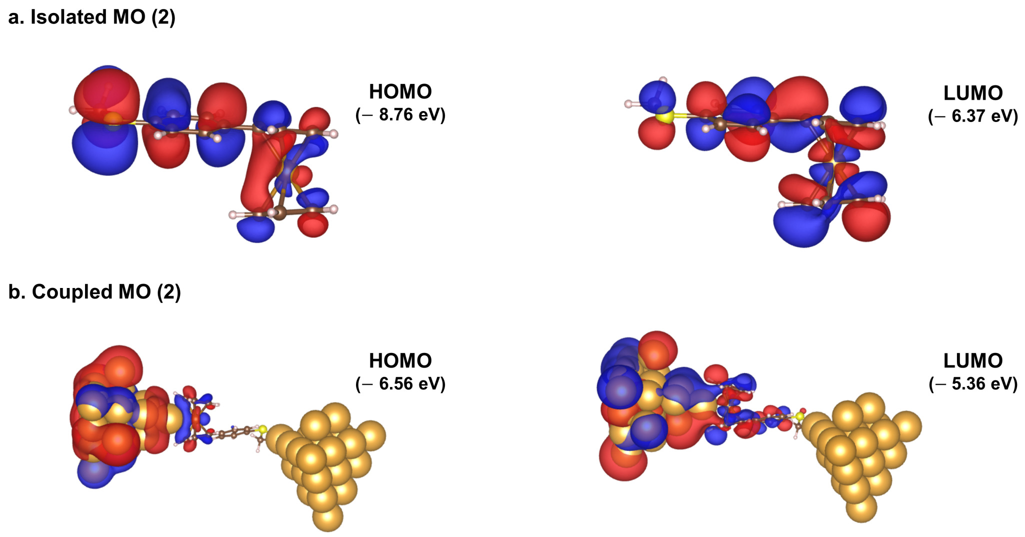

Supplementary Fig. 13. (a) Molecular orbitals of isolated molecule **2**. (b) Scattering states of the **2H** junction, that contribute to transmission at E_F. Each frontier orbital energy is calculated relative to the vacuum level. E_F is – 6.31 eV relative to vacuum.

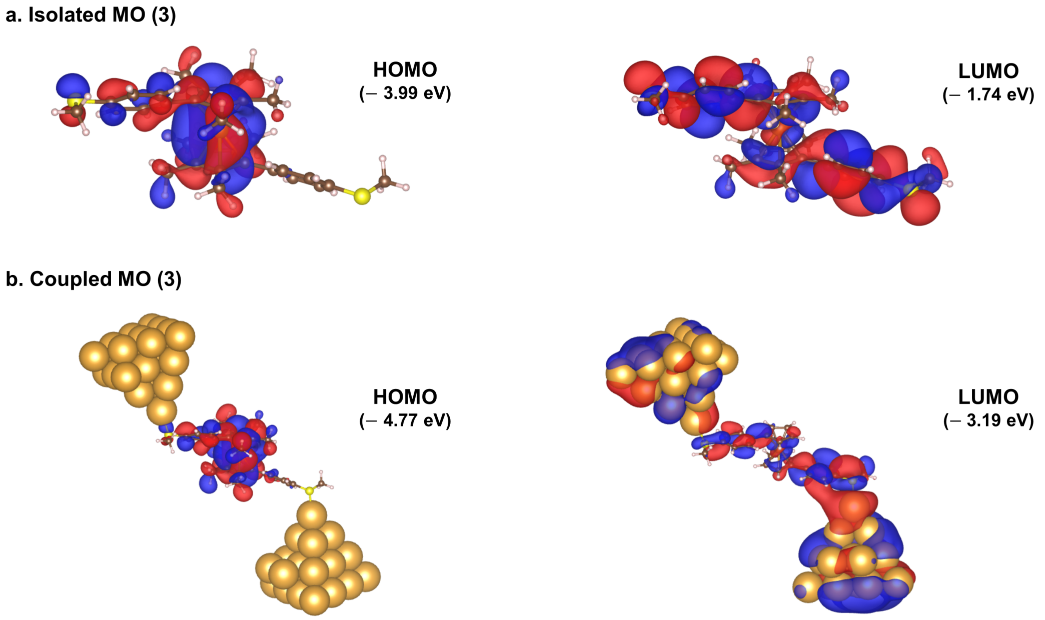

Supplementary Fig. 14. (a) Molecular orbitals of isolated molecule **3**. (b) Scattering states of the **3L** junction, that contribute to transmission at E_F. Each frontier orbital energy is calculated relative to the vacuum level. E_F is – 4.52 eV relative to vacuum.

Transmission calculations for 1H junction

The transmission of **1H** is calculated using the same procedures as explained for **2H** in the main manuscript. The resulting net charge on **1** within the junction is +0.650, indicating the **1H** junction is in an oxidized state. The relaxed geometry for **1H** and **2H** are shown in Supplementary Fig. 15a, b. An additional thioanisole group in **1** significantly changes the direction of the electrode linked to Fe, resulting in the conductance difference between **1H** and **2H** junctions. Despite the shared electron pathway of **1H** and **2H**, they exhibit distinct conductance values.

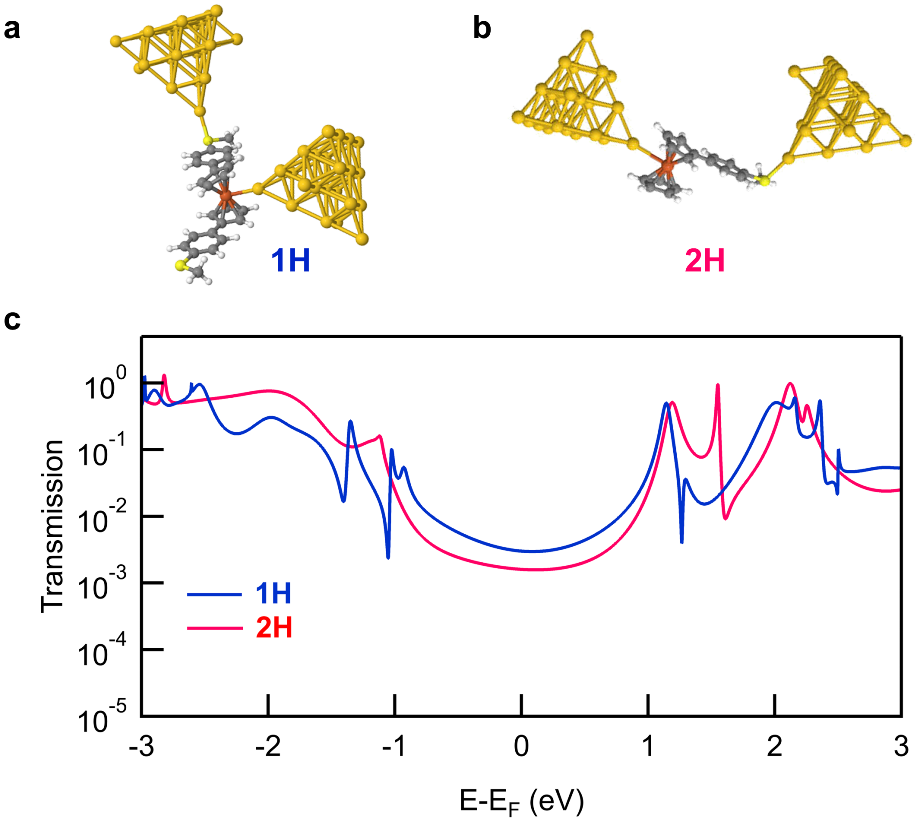

Supplementary Fig. 15. (a) The relaxed junction geometries for (a) **1H** and (b) **2H**. Dark grey, light grey, red, yellow, and gold spheres represent C, H, Fe, S, Au atoms, respectively. (c) Calculated transmission functions for **1H** and **2H**.

Transmission calculations for 3L junction

The transmission function for **3L** and the [**3L**]$^+$ junctions are shown in Supplementary Fig. 16c. The oxidized molecule transmission is determined by adding eight-point charges, each consisting of - 0.125 unit of charge, around the molecule **3** as shown in Supplementary Fig. 16b.[15] The net charge on [**3L**]$^+$ is +0.636. The spin-up and spin-down channels have similar transmissions at E_F. The transmission calculation shows that **3L** has a higher conductance than [**3L**]$^+$ in agreement with the experimental data shown in Fig. 2c. As the HOMO resonance in the spin-down channel is close to E_F, conductance is likely to be sensitive to the bias voltage used in the measurement.

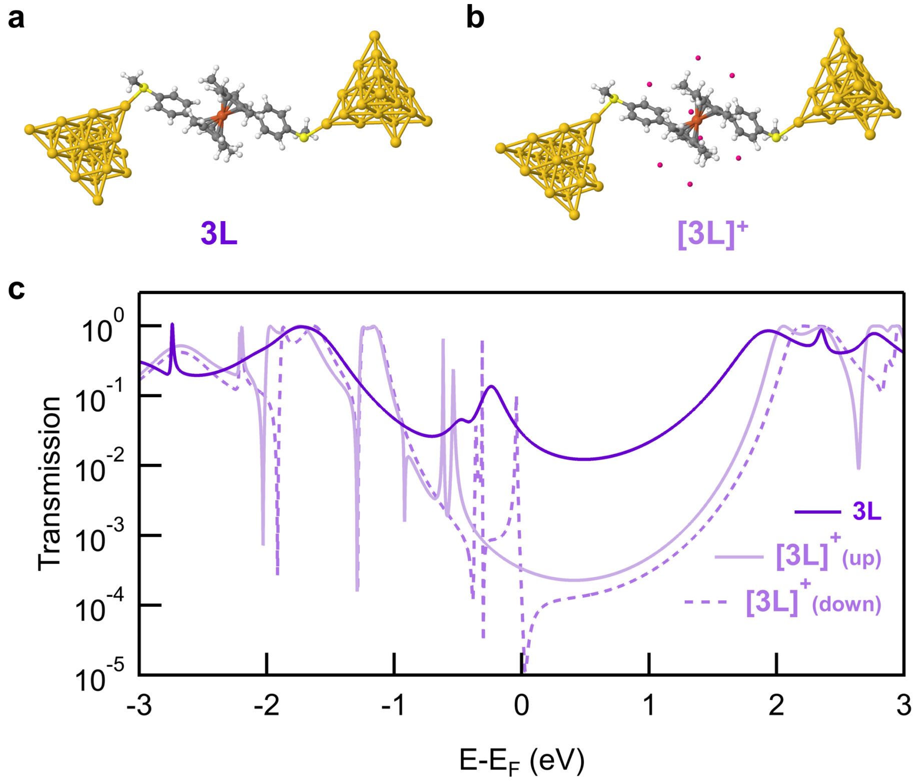

Supplementary Fig. 16. The relaxed junction geometries for (a) **3L** and (b) [**3L**]$^+$. Dark grey, light grey, red, yellow, and gold spheres represent C, H, Fe, S, Au atoms, respectively. (c) Calculated transmission functions for **3L**, [**3L**]$^+$ (spin-up) and, [**3L**]$^+$ (spin-down).

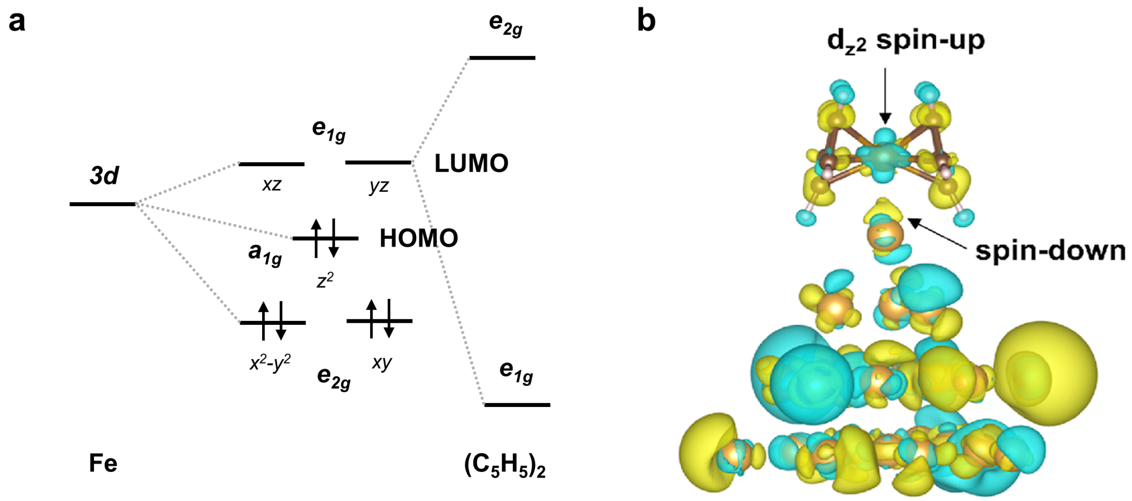

Supplementary Fig. 17. (a) The molecular orbital diagram of ferrocene with D_{5h} symmetry.[16,17] The HOMO orbital is an Fe atom based d_{z^2} orbital. Once the ferrocene is oxidized, the singly occupied d_{z^2} orbital (SOMO) will participate in the bond formation with the Au. We note that the π-orbitals of Cp rings do not contribute the HOMO (or SOMO) d_{z^2} orbitals. (b) Calculated spin density distribution of the Fe-Au contact, showing that the d_{z^2} orbital is located on the iron center. This is consistent with the molecular orbital diagram, suggesting that the iron center is the most favorable site for binding to gold electrode in its oxidized state. Light blue and yellow regions denote spin-up and spin-down densities, respectively. Those antiparallel spins interact with each other, forming the Fe-Au bond. To make the spin density visible, the isosurface value is set to be 10^{-7}.

Supplementary Method - Synthetic Details and NMR spectra

All reactions were conducted in oven-dried glassware under a nitrogen or argon atmosphere, though no special precautions were taken to exclude air or moisture during workup and purification. Negishi conditions were based on previous reports.[1,2] Zinc chloride was dried immediately prior to use by heating under vacuum at 140°C overnight.[3] Anhydrous and deoxygenated THF was prepared by sparging with nitrogen and elution through a dual-column solvent purification system (MBraun SPS or Pure Process Technologies, Nashua, NH, USA). All other materials were commercially available and used as received. ^{1}H and ^{13}C{^{1}H} were recorded at room temperature on a Bruker Avance III 400, 400SL (400 MHz) or Varian VNMRS 400 (400 MHz), or VNMRS 600 (600 MHz) spectrometer. ^{1}H NMR data recorded in $CDCl_3$ and CD_2Cl_2 is referenced to residual internal C*H*Cl_3 (δ 7.26 ppm) and C*H*DCl_2 (δ 5.32) solvent signals. ^{13}C{^{1}H} NMR data recorded in $CDCl_3$ and CD_2Cl_2 is referenced to internal *C*DCl_3 (δ 77.16 ppm) and *C*D_2Cl_2 (δ 53.84) solvent signals. Mass spectrometry analyses were conducted at the Mass Spectrometry Facility, Chemistry Department, Columbia University, New York using a Waters XEVO G2-XS QToF mass spectrometer, or at the University of Southern California using an Agilent 6545 QTOF mass spectrometer fitted with an atmospheric pressure electrospray ionization source (Dual AJS ESI).

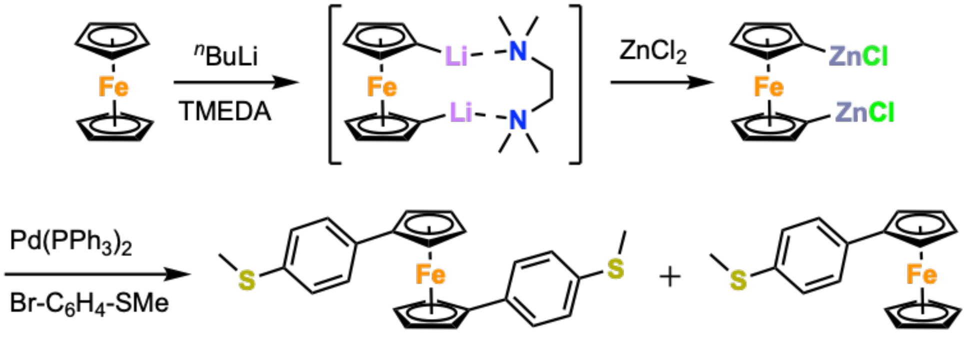

1 **2**

Supplementary Fig. 18. Synthetic route to **1** and **2** (TMEDA = N,N,N’,N’-tetramethylethanediamine).

*1,1'-Bis(4-thiomethoxyphenyl)ferrocene (**1**) and 1-(4-Thiomethoxyphenyl)ferrocene (**2**)*

This method was adapted from the previously reported synthesis of **1**.[4] TMEDA (1.7 mL, 11 mmol) and 2.5 M *n*-BuLi in hexanes (4.8 mL, 12 mmol) were added to a stirred suspension of ferrocene (0.988 g, 5.31 mmol) in *n*-hexane (20 mL) held at 0°C (ice bath). After stirring overnight to room temperature, the majority of solvent was removed by cannula filtration and the orange slurry dissolved in THF (16 mL) at 0°C. A freshly prepared solution of ~0.98 M ZnCl in THF (11.5 mL, ~11.3 mmol) was added, and the mixture was stirred at room temperature for 2.5 h. $Pd(PPh_3)_4$ (0.779 g, 0.674 mmol) and 4-bromothioanisole (2.427 g, 11.95 mmol) were added against nitrogen and the reaction was stirred at 60°C for 18 h. After cooling to room temperature, solvent was removed *in vacuo*. The crude product was pre-absorbed on silica and purified by column chromatography, eluting with CH_2Cl_2-hexanes (0:100→30:70 v/v). The crude material obtained after evaporation of fractions from the first orange band (eluting with 10% CH_2Cl_2 in hexanes), was recrystallized from CH_2Cl_2/hexanes to provide **2** as an orange solid (0.1574 g, 10%). ^{1}H NMR (400 MHz, $CDCl_3$): δ (ppm) 2.50 (s, 3H, -SC*H*$_3$), 4.04 (s, 5H, Cp-*H*), 4.30 (pseudo-t, 2H, *J* = 2.0 Hz, Cp-H), 4.61 (pseudo-t, 2H, *J* = 2.0 Hz, Cp-*H*), 7.19 (d, 2H, *J* = 8.7 Hz, Ar-*H*), 7.42 (m, 2H, *J* = 8.6 Hz, Ar-*H*). ^{13}C{^{1}H} NMR (150 MHz, $CDCl_3$): δ (ppm) 16.25 (2C, -S*C*H$_3$) 66.41 (2C, Cp *C*-H), 69.02 (2C, Cp *C*-H), 69.72 (5C, Cp *C*-H), 85.06 (1C, Cp *C*-Ar), 126.63 (2C, Ar *C*-H), 127.00 (2C, Ar *C*-H), 135.50 (1C, Ar *C*-R), 136.44 (1C, Ar *C*-R). HR-MS (ESI+): m/z 308.0358 ($[M+H]^+$ calc. for $C_{17}H_{16}FeS$: 308.0322). The crude material obtained after evaporation of fractions from the second orange band was recrystallized from CH_2Cl_2/hexanes to provide **1** as an orange solid (0.7547 g, 33%). Spectroscopic data was consistent with previous reports.[4] ^{1}H NMR (400 MHz, $CDCl_3$): δ (ppm) 2.51 (s, 6H, -SC*H*$_3$), 4.21 (pseudo-t, 4H, Cp-*H*), 4.44 (pseudo-t, 4H, Cp-*H*), 7.07 (d, 4H, Ar-*H*), 7.17 (d, 4H, Ar-*H*).

Cp-SMe

This method was adapted from the reported syntheses of anisole and thioanisole analogues.[5,6] A solution of 1.6 M *n*-BuLi in hexanes (5 mL, 8 mmol) was added portionwise to a solution of 4-bromothioanisole (1.464 g, 7.206 mmol) in diethyl ether (15 mL) held at 0°C (water-ice bath). The cooling bath was removed and the reaction stirred at room temperature for 2 h, whereby solvent was removed *in* vacuo without exposing the system to air. The white solid residue

was cooled to −78°C (dry ice-acetone bath) and THF (13 mL) was added. A solution of 2,3,4,5-tetramethyl-2-cyclopentenone (1.2 mL, 8 mmol) in THF (6 mL) was cooled to −78°C and added to the stirred suspension of 4-lithiothioanisole. After warming to room temperature overnight, a saturated aqueous solution of ammonium chloride (18 mL) was added to the orange solution to quench the reaction. The organic layer was separated, the aqueous component further extracted with diethyl ether (28 mL), and the combined extracts were dried over $MgSO_4$. After filtration and solvent removal, the pale-yellow oil was re-dissolved in CH_2Cl_2 (12 mL). A catalytic quantity of *p*-toluenesulfonic acid (~0.1 g) was added and the mixture was stirred at room temperature for 1 h. Solvent was removed and the residue purified by column chromatography (alumina Brockmann grade II; hexanes/CH_2Cl_2, 1:0 → 9:1), providing **Cp-SMe** as a yellow-orange solid (0.350 g, 20%). ^1H NMR (400 MHz, CD_2Cl_2): δ (ppm) 0.92 (d, 3H, J = 7.7 Hz, Cp-CH_3), 1.85 (quint., 3H, J = 1.2 Hz, Cp-CH_3), 1.92 (br. s, 3H, Cp-CH_3), 2.01 (d, 3H, J = 1.9 Hz, Cp-CH_3), 2.49 (s, 3H, -SCH_3), 3.16 (m, 1H, Cp-*H*), 7.17 (d, 2H, J = 8.6 Hz, Ar-*H*), 7.25 (d, 2H, J = 8.6 Hz, Ar-*H*). $^{13}C\{^1H\}$ NMR (100 MHz, CD_2Cl_2): δ (ppm) 11.21, 12.04, 12.95, 15.11, 16.26, 50.30, 126.96, 129.20, 134.57, 135.43, 135.46, 137.70, 141.12, 142.29. HR-MS (ESI+): m/z 244.1290 ($[M]^+$ calc. for $C_{16}H_{20}S$: 244.1286).

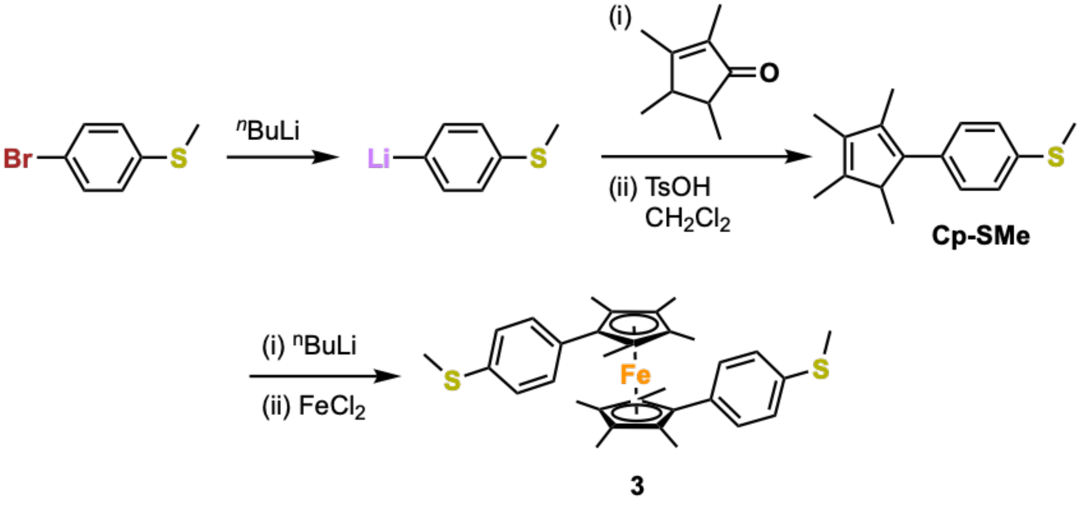

Supplementary Fig. 19. Synthetic route to **3**.

1,1'-Bis(4-thiomethoxyphenyl)octamethylferrocene (**3**)

This method was adapted from an analogous procedure.[7] A solution of **Cp-SMe** (0.149 g, 0.610 mmol) in THF (4 mL) was stored over activated molecular sieves (3Å, ~0.5 g) for 1 h to remove the majority of adventitious water. This solution was cannula transferred to a separate flask, washing through with additional THF (2 mL). After cooling to 0°C (water-ice bath), a solution of 1.6 M *n*-BuLi in hexanes (0.39 mL, 0.62 mmol) was added dropwise. This solution was cannula transferred to a flask containing $FeCl_2$ (0.035 g, 0.28 mmol) in THF (2 mL), and the reaction mixture heated at reflux for 2 h. After cooling, methanol (0.5 mL) was added to the resulting orange-brown solution to quench residual organolithium species and solvent was removed. The dried residue was subjected to column chromatography (alumina Brockmann grade II; CH_2Cl_2/MeOH, 1:0 → 95:5) where a significant proportion changed color from orange to green, indicating the formation of oxidized ferrocenium species. Eluting green fractions were treated with excess sodium ascorbate[8] in methanol to provide the reduced orange ferrocene product, whereby solvent was removed and the dried residue extracted into hexanes. Selected fractions were combined to provide **3** as an orange solid (0.020 g, 15%) after evaporation of solvent. ^{1}H NMR (400 MHz, CD_2Cl_2): δ (ppm) 1.71 (s, 12H, Cp-CH_3), 1.76 (s, 12H, Cp-CH_3), 2.47 (s, 6H, -SCH_3), 7.03 (d, 4H, J = 8.6 Hz, Ar-*H*), 7.09 (d, 4H, J = 8.6 Hz, Ar-*H*). ^{13}C{^{1}H} NMR (100 MHz, CD_2Cl_2): δ (ppm) 9.87 (4C, Cp-*C*H$_3$), 11.03 (4C, Cp-*C*H$_3$), 16.19 (2C, -S*C*H$_3$), 78.86 (4C, Cp *C*-Me), 80.79 (4C, Cp *C*-Me), 85.92 (2C, Cp *C*-Ar), 126.05 (4C, Ar *C*-H), 131.63 (4C, Ar *C*-H), 134.42 (2C, Ar *C*-R), 135.37 (2C, Ar *C*-R). HR-MS (ESI+): m/z 542.1766 ($[M+H]^+$ calc. for $C_{32}H_{38}FeS_2$: 542.1764).

NMR Spectra

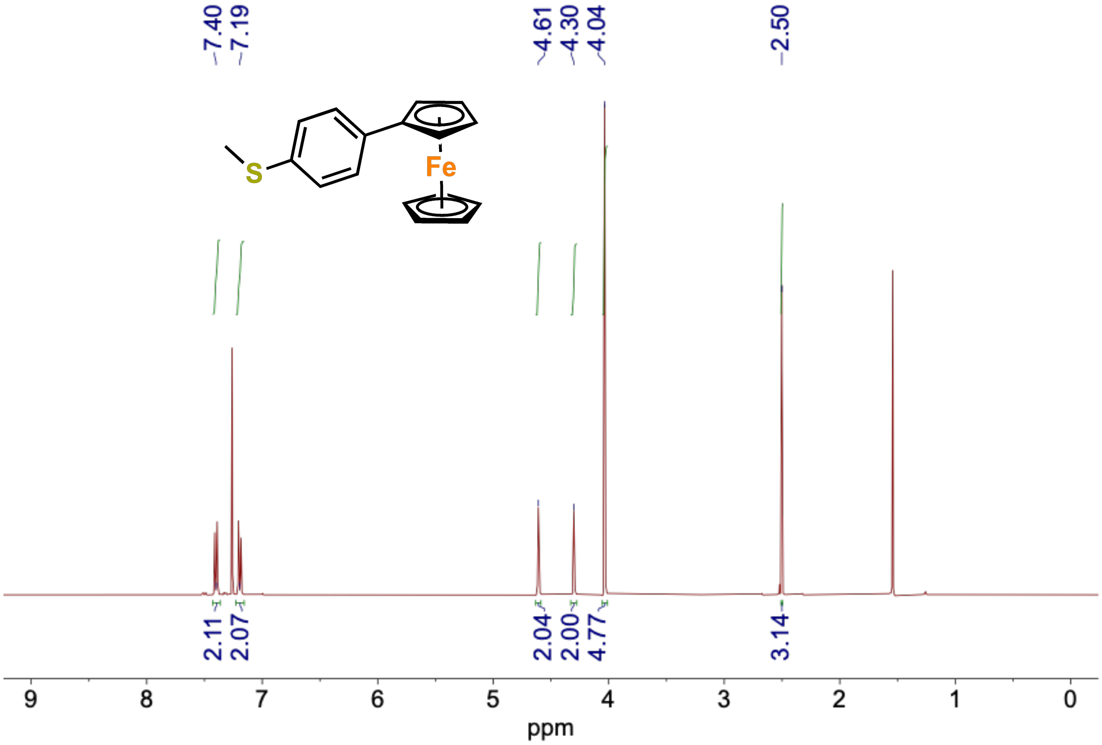

Supplementary Fig. 20. ^{1}H NMR (400 MHz) spectrum of **2** in $CDCl_3$.

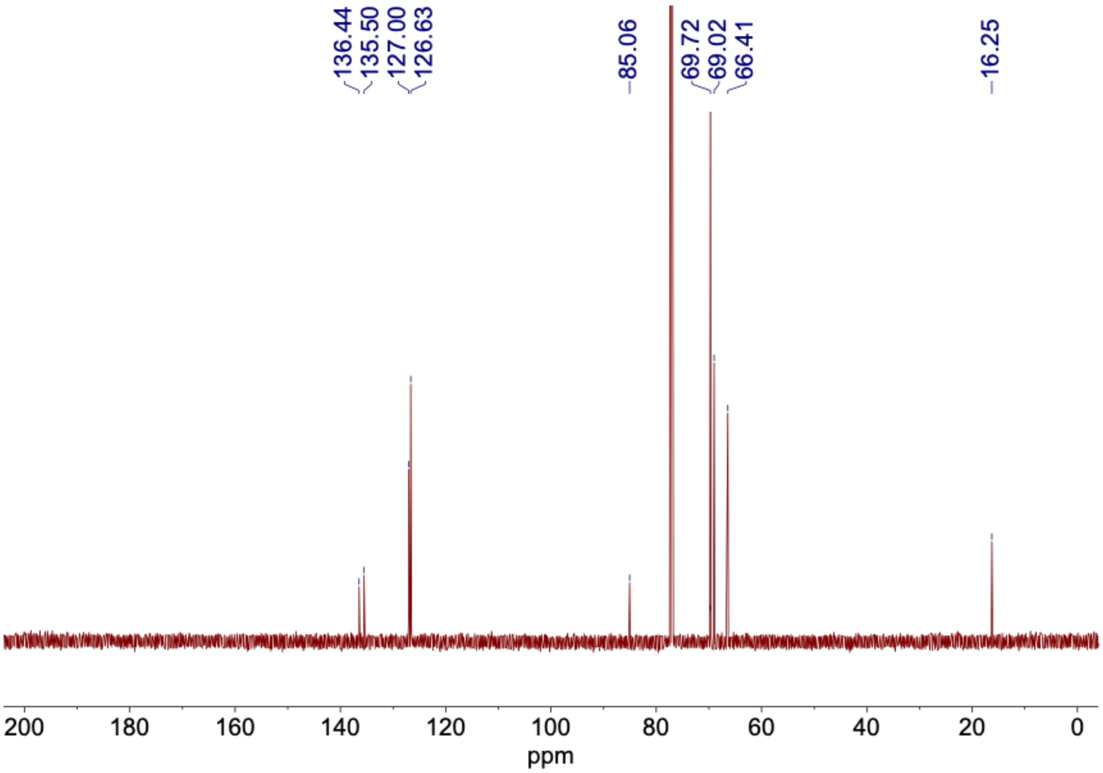

Supplementary Fig. 21. $^{13}C\{^1H\}$ (150 MHz) NMR spectrum of **2** in $CDCl_3$.

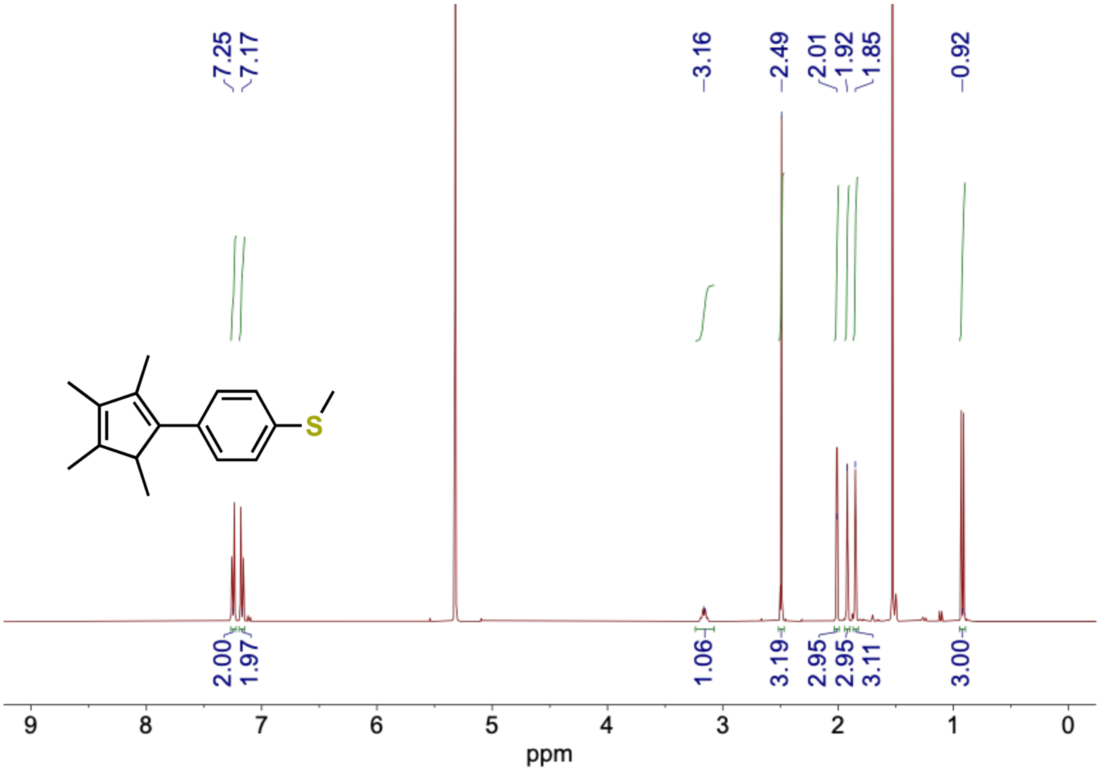

Supplementary Fig. 22. ^{1}H NMR (400 MHz) spectrum of **Cp-SMe** in CD_2Cl_2.

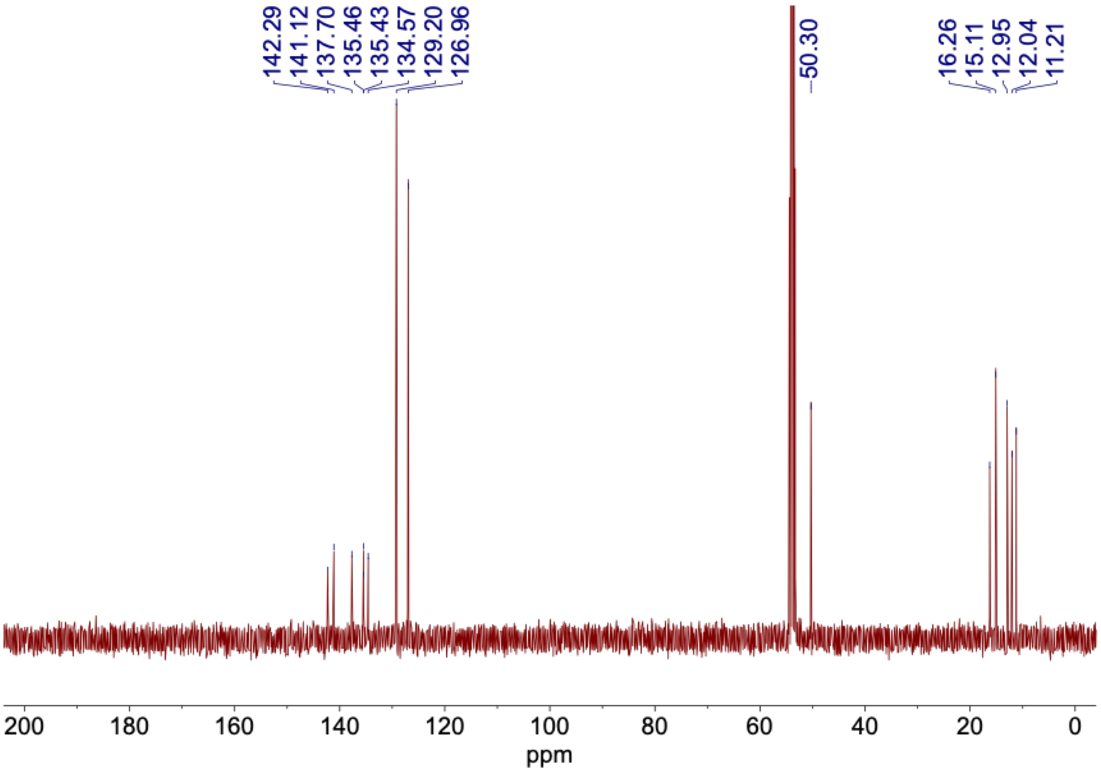

Supplementary Fig. 23. $^{13}C\{^{1}H\}$ NMR (100 MHz) spectrum of **Cp-SMe** in CD_2Cl_2.

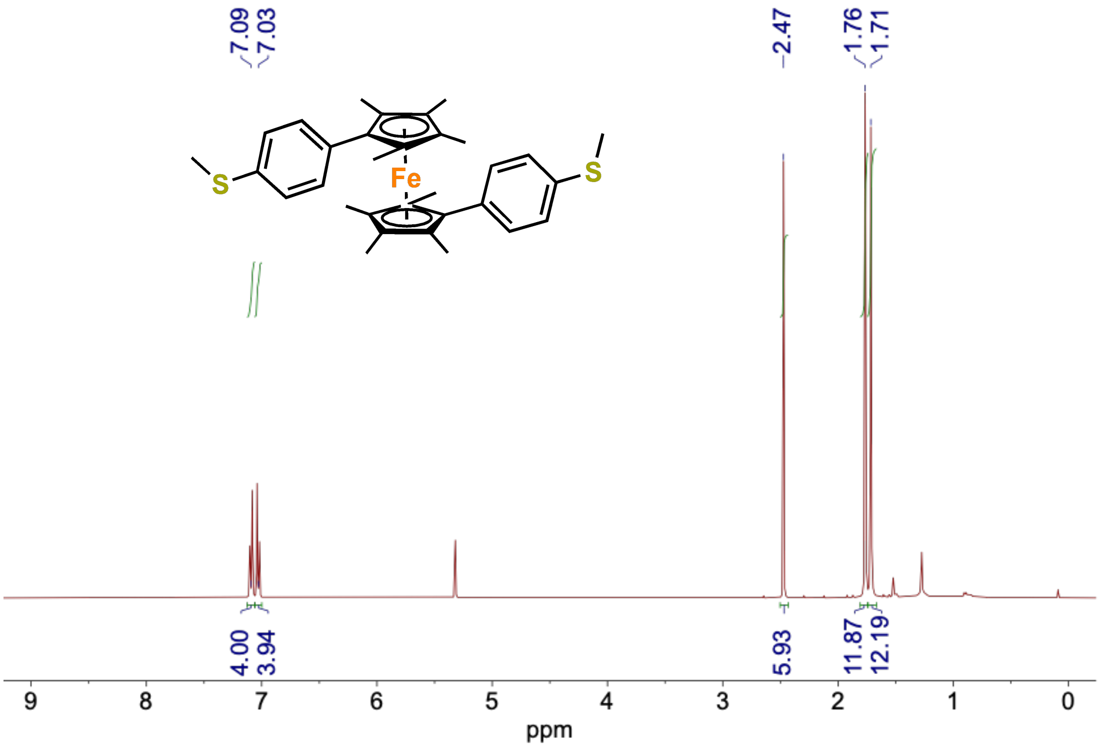

Supplementary Fig. 24. ^{1}H NMR (400 MHz) spectrum of **3** in CD_2Cl_2.

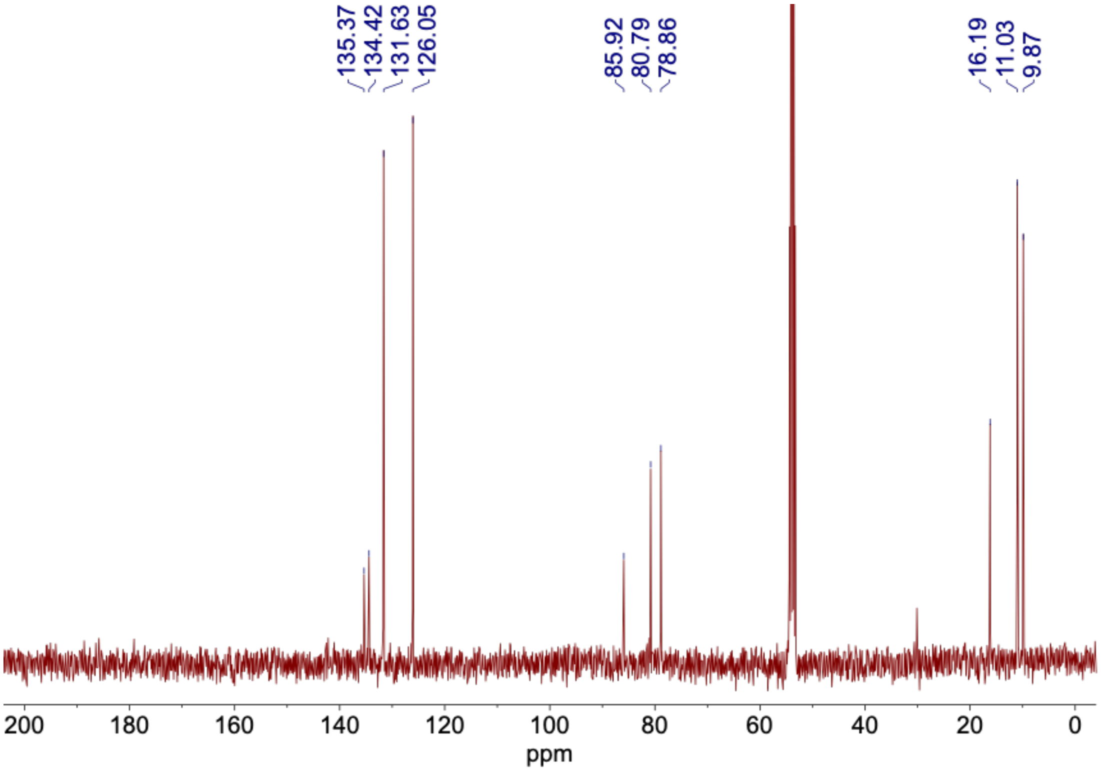

Supplementary Fig. 25. $^{13}C\{^1H\}$ NMR (100 MHz) spectrum of **3** in CD_2Cl_2.

References

1 Braunschweig, H., Damme, A., Hammond, K. & Mager, J. Synthesis and structure of new [3] silametallocenophanes of group 8 metals. *Organometallics* **31**, 6317-6321 (2012).

2 Enders, M., Kohl, G. & Pritzkow, H. Novel Heterobimetallic Compounds with Metal–Metal Bonds: The Use of Quinolyl-Substituted Metallocenes as Tridentate Ligands. *Organometallics* **21**, 1111-1117 (2002).

3 Cotton, H. K., Huerta, F. F. & Bäckvall, J. E. Highly Selective Negishi Cross-Coupling Reaction of a Zinc-Metallated Ferrocenyl p-Tolyl Sulfoxide: New Chiral Ferrocene-Based Quinone Ligands. *European Journal of Organic Chemistry* **2003**, 2756-2763 (2003).

4 Camarasa-Gómez, M. *et al.* Mechanically Tunable Quantum Interference in Ferrocene-Based Single-Molecule Junctions. *Nano Letters* **20**, 6381-6386 (2020).

5 Kim, S. K., Kim, H. K., Lee, M. H., Yoon, S. W. & Do, Y. Novel synthetic strategy for developing an isospecific unbridged metallocene system for propylene polymerization. *Chem. Eur. J.* **13**, 9107-9114 (2007).

6 Piou, T. *et al.* Correlating reactivity and selectivity to cyclopentadienyl ligand properties in Rh (III)-catalyzed C–H activation reactions: an experimental and computational study. *J. Am. Chem. Soc.* **139**, 1296-1310 (2017).

7 Threlkel, R. S. & Bercaw, J. E. A convenient synthesis of alkyltetramethylcyclopentadienes and phenyltetramethylcyclopentadiene. *J. Organomet. Chem.* **136**, 1-5 (1977).

8 Cunningham, K. L. & McMillin, D. R. A simple route to FeCpCp′. *Polyhedron* **15**, 1673-1675 (1996).

9 Capozzi, B. *et al.* Tunable charge transport in single-molecule junctions via electrolytic gating. *Nano Lett.* **14**, 1400-1404 (2014).

10 Capozzi, B. *et al.* Mapping the transmission functions of single-molecule junctions. *Nano letters* **16**, 3949-3954 (2016).

11 Grimme, S. Semiempirical GGA-type density functional constructed with a long-range dispersion correction. *Journal of computational chemistry* **27**, 1787-1799 (2006).

12 Ricci, F. & Profeta, G. van der Waals interaction in iron-chalcogenide superconductors. *Phys. Rev. B* **87**, 184105 (2013).

13 Amft, M., Lebègue, S., Eriksson, O. & Skorodumova, N. V. Adsorption of Cu, Ag, and Au atoms on graphene including van der Waals interactions. *Journal of Physics: Condensed Matter* **23**, 395001 (2011).

14 Schäfer, K. M., Reinders, L., Fiedler, J. & Ringenberg, M. R. Twisting and Tilting 1, 1′-Bis (dialkylphosphino) ferrocene Bound to Low Valent Tricarbonylmaganese (I to− I). *Inorganic Chemistry* **56**, 14688-14696 (2017).

15 Li, L. *et al.* Highly conducting single-molecule topological insulators based on mono- and di-radical cations. *Nat. Chem.* **14**, 1061-1067 (2022).

16 Rahnamaye Aliabad, H. & Chahkandi, M. Comprehensive SPHYB and B3LYP-DFT Studies of Two Types of Ferrocene. *Zeitschrift für anorganische und allgemeine Chemie* **643**, 420-431 (2017).

17 Yamaguchi, Y. & Kutal, C. Efficient photodissociation of anions from benzoyl-functionalized ferrocene complexes. *Inorganic chemistry* **38**, 4861-4867 (1999).
